# A Super TLR Agonist to Improve Efficacy of Dendritic Cell Vaccine in Induction of Anti-HCV Immunity

**DOI:** 10.1371/journal.pone.0048614

**Published:** 2012-11-07

**Authors:** Bangxing Hong, Sung-Hyung Lee, Xiao-Tong Song, Lindsey Jones, Keigo Machida, Xue F. Huang, Si-Yi Chen

**Affiliations:** 1 Norris Comprehensive Cancer Center, Department of Molecular Microbiology & Immunology, University of Southern California Keck School of Medicine, Los Angeles, California, United States of America; 2 Center for Cell and Gene Therapy, Department of Pediatrics, Baylor College of Medicine, Houston, Texas, United States of America; The Ohio State University, United States of America

## Abstract

Persistent infections caused by pathogens such as hepatitis C virus are major human diseases with limited or suboptimal prophylactic and therapeutic options. Given the critical role of dendritic cell (DC) in inducing immune responses, DC vaccination is an attractive means to prevent and control the occurrence and persistence of the infections. However, DCs are built-in with inherent negative regulation mechanisms which attenuate their immune stimulatory activity and lead to their ineffectiveness in clinical application. In this study, we developed a super DC stimulant that consists of a modified, secretory Toll-like Receptor (TLR)-5 ligand and an inhibitor of the negative regulator, suppressor of cytokine sinaling-1 (SOCS1). We found that expressing the super stimulant in DCs is drastically more potent and persistent than using the commonly used DC stimuli to enhance the level and duration of inflammatory cytokine production by both murine and human DCs. Moreover, the DCs expressing the super stimulant are more potent to provoke both cellular and humoral immune responses against hepatitis C virus (HCV) antigen *in vivo*. Thus, the strategy capable of triggering and sustaining proinflammatory status of DCs may be used to boost efficiency of DC vaccine in preventing and combating the persistent infection of HCV or other chronic viruses.

## Introduction

Intensive efforts have been devoted to develop Toll-like receptor (TLR) agonists to improve vaccine efficacy, since antigen-presenting cells (APCs), such as dendritic cells (DCs), use TLRs to sense conserved structural moieties of pathogens for the activation of proinflammatory signaling cascades, and innate/adaptive immune responses [1], [2], [3]. However, pathogens associated with chronic infections such as human immunodeficiency virus (HIV) and hepatitis C virus (HCV) are refractory to the immune responses that are induced either by vaccination or natural infection of pathogens that contain TLR ligands themselves. Thus, there is an urgent need to develop novel approaches capable of stimulating more potent immune responses that are superior to the natural immunity.

Our recent studies demonstrated that the suppressor of cytokine signaling (SOCS) 1, a key negative regulator of Janus kinase (JAK)/signal transducer and activator of transcription (STAT) signaling [4] and TLR signaling, inhibited the maturation, inflammatory cytokine production, and immunostimulatory potency of DCs, functioning as a critical antigen presentation attenuator [5], [6]. We further demonstrated that silencing of SOCS1 drastically enhanced the sensitivity of DCs to stimulation with TLR agonists. However, inhibition of SOCS1 alone is insufficient to fully activate DCs, since proinflammatory stimuli such as TLR agonists are required to initiate proinflammatory signaling cascades and produce various proinflammatory cytokines. The bacterial filament protein flagellin (FliC) activates APCs via its interaction with the surface TLR5 [7], [8]. TLR5 is expressed by a variety of cells including monocytes, DCs, and epithelial cells. TLR5 engagement by flagellin activates the MyD88-dependent signaling pathway, which leads to the activation of NF-κB and MAPKs, ultimately inducing the maturation of APCs and the secretion of proinflammatory cytokines and chemokines. Recombinant flagellin-antigen fusion proteins have been shown to enhance the immunogenicity of antigens and induce potent T cell and antibody responses in mice and monkeys [9], [10], [11], [12], [13], [14]. Moreover, DNA vectors coexpressing flagellin significantly enhanced antigen-specific T cell and antibody responses in mice [15]. Thus, in this study we designed and tested a super agonist of TLR, designated as super-TLR-agonist, consisting of FliC and a small inhibitory RNA of SOCS1, for triggering and sustaining TLR signaling and cytokine signaling cascades in DCs. The expression of the super-TLR-agonist in DCs is found more potent and persistent than using commercial TLR agonist as a maturation agent in stimulating the level and duration of inflammatory cytokine production by both murine and human DCs. Moreover, the super-TLR-agonist-expressed DCs display a superior ability to activate HCV antigen-specific cellular and humoral immune responses.

## Results

### The Expression of SOCS1-shRNA (shS1)/FliC Rather than shS1 or FliC Enhances the Levels and Duration of Proinflammatory Cytokine Production by DCs

DCs activate T lymphocytes by providing three general types of signals: MHC-restricted antigenic peptides, costimulatory molecules, and cytokines/chemokines. To test whether expressing shS1 nand secretory FliC in bone marrow-derived DCs (BMDCs) results in enhanced and prolonged production of proinflammatory cytokines/chemokines and elevated expression of costimulatory molecules and MHC molecules, we constructed and produced a recombinant replication-deficient adenoviral (Ad) vector coexpressing shS1 and a modified, secretory FliC [7] genetically linked with a signal leader sequence, designated as Ad-shS1/FliC, and a panel of recombinant Ad vectors expressing shS1, shGFP, or secretory FliC alone (**Fig. S1A**). Recombinant Ad vectors were used because they are the most efficient of gene delivery systems for transient gene expression in murine and human DCs and are able to modestly stimulate DCs [16], [17]. We first used increasing titers of the adenoviral vector [multiplicity of infection (MOI) = 0, 50, 100, 250, or 500] to transduce BMDCs and identified that the adenoviral vector at an MOI of 250 effectively transduced ∼70–80% of BMDCs as indicated by GFP expression in the Ad-shGFP-transduced sample, but did not cause any significant cell death (data not shown). The ability to downregulate SOCS1 of Ad-shS1/FliC and Ad-shS1 was confirmed by quantitative RT-PCR (qRT-PCR) (**Fig. S1B**). High levels of FliC protein were produced by BMDCs transduced with Ad-shS1/FliC or Ad-FliC, as tested by Western Blot (**Fig. S1C**).

**Figure 1:**
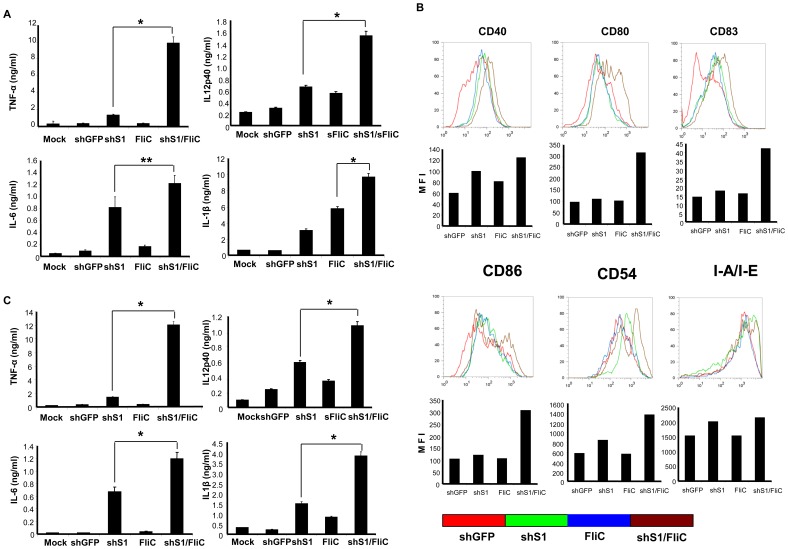
The expression of shS1/FliC rather than shS1 or FliC enhances the levels and duration of proinflammatory cytokine production by DCs. Murine BMDCs were transduced with the recombinant Ad vectors at an MOI of 250. A, 24 h later, culture media were collected for evaluation of the representative cytokines by ELISA. B, cells were analyzed for expression of costimulatory and MHC class-II molecules by flow cytometry. C, 24 h later, cultures were washed and replaced with fresh medium that does not contain stimuli. The concentrations of representative cytokines in culture medium 3 days after washing were examined by ELISA. Data are representative of three repeated experiments. *p<0.01; **p<0.05.

We tested whether Ad-shS1/FliC transduction is more potent than Ad-shS1 or Ad-FliC transduction in enhancing expression of proinflammatory cytokines, costimulatory molecules, and MHC molecules in BMDCs as well as a DC cell line and a macrophage cell line. Murine BMDCs, J774A.1 macrophages, and D2SC/1 DCs that express surface TLR5 (**Fig. S2**) were used for this study. We found that dramatically higher levels of TNF-α and IL-12p40, and significantly higher levels of IL-6 and IL-1β were produced by Ad-shS1/FliC-transduced BMDCs compared to those by Ad-shS1 or Ad-FliC-transduced BMDCs (**Fig. 1A**). Higher levels of costimulatory molecules such as CD80, CD83 and CD86 were also observed in Ad-shS1/FliC-BMDCs (**Fig. 1B**). However, the expression of CD40, CD54, and MHC class-II molecule I-A/I-E did not show any significant difference among the differently transduced BMDCs (**Fig. 1B**). A similar profile of cytokine production was obtained in adenoviral-transduced J774A.1 macrophages (**Fig. S3**) and D2SC/1 DCs (data not shown). Thus, these results indicate a marked synergistic effect of SOCS1 silencing and TLR stimulation in promoting proinflammatory status in shS1/FliC-expressed DCs. Our previous studies revealed that SOCS1-silenced DCs did not express higher levels of the costimulatory molecules in response to LPS stimulation [5], [18]. The discrepancy may be owed to a different TLR (TLR5 vs. TLR4) engaged in activation of Ad-shS1/FliC-transduced DCs in this study.

Since duration and intensity of antigen presentation is important in determining magnitude and memory of adaptive immune responses [19], we compared the duration of inflammatory cytokine production by the transduced DCs. BMDCs were transduced with different adenoviral vectors, and then washed and added with fresh culture medium 24 hrs after the transduction. The cytokine concentrations in culture medium were examined 72 hours after washing. **Fig. 1C** shows that Ad-shS1/FliC-DCs produced higher levels of inflammatory cytokines such as TNF-α, IL-12p40, IL-6, and IL-1β after washing. These data showed expression of shS1/FliC endows DCs with a unique ability to enhance and prolong their inflammatory cytokine production.

### Expressing shS1/FliC is More Potent than Using TLR Agonist as a Maturation Agent to Enhance the Levels and Duration of Proinflammatory Cytokine Production by DCs

**Figure 2:**
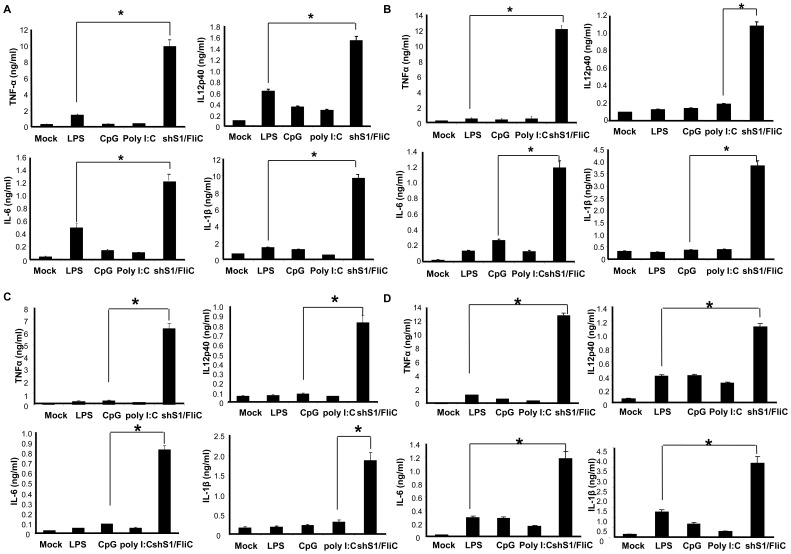
The expression of shS1/FliC is more potent than using TLR agonist as a maturation agent to enhance the levels and duration of proinflammatory cytokine production by DCs. Murine BMDCs were transduced with Ad-shS1/FliC or stimulated with commonly used TLR agonist LPS (100 ng/ml), CpG (1 µM), or Poly I:C (1 µg/ml), respectively. **A,** 24 h later, culture media were collected for evaluation of the representative cytokines by ELISA. **B,** 24 h later, cultures were washed and replaced with fresh medium that does not contain the corresponding stimuli. The concentrations of representative cytokines were examined by ELISA in culture medium 72 hr after the wash. **C&D**, 24 h later, cultures were washed and replaced with fresh medium that contains the corresponding stimuli or not (for Ad-shS1/FliC-transduced DCs). The concentrations of representative cytokines were examined by ELISA 24 hr (**C**) or 72 hr (**D**) after the wash. Data are representative of three repeated experiments. *p<0.01.

Numerous TLR agonists, such as imiquimod, polyI:C, and CpG, have been developed for promoting immunostimulatory activity of DCs [3], [20]. We then examined whether Ad-shS1/FliC transduction is more potent than using commercial TLR agonist as a maturation agent in enhancing and prolonging DCs to express proinflammatory cytokines. **Fig. 2A** shows that Ad-shS1/FliC transduction was more potent than commonly used TLR agonists (LPS, CpG, or PolyI:C) in stimulating DCs to express the proinflammatory cytokines such as TNF-α, IL-12p40, IL-6, and IL-1β. Furthermore, we compared the duration of the inflammatory cytokine production by DCs treated with different stimuli. BMDCs were transduced with Ad-shS1/FliC vector or stimulated with TLR agonists for 24 hr, then washed and cultured for another 24–72 hr with fresh culture medium that does or does not contain stimuli. The cytokine concentrations in culture medium were examined. Result shows that Ad-shS1/FliC-DCs kept producing high levels of the inflammatory cytokines after washing. In contrast, TLR agonist-DCs were unable to actively produce the cytokines without re-stimulation (**Fig. 2B**) or with 24 hr re-stimulation of TLR ligand (**Fig. 2C**), which is consistent with the published studies that the TLR agonist-matured DCs are tolerant to re-stimulation of TLR agonist [21], [22]. **Fig. 2D** shows that TLR agonist-DCs could recover cytokine production in response to 72 hr re-stimulation of TLR ligand, but the cytokine levels was much lower than that produced by Ad-shS1/FliC-DCs.

TLR agonists were reported to have synergic stimulatory activity [23]. Hence, the shS1/FliC expression was compared with the synergic stimulation of TLR agonists in potentiating inflammatory status and duration of DCs. Results show that Ad-shS1/FliC transduction was ∼50% stronger in facilitating DCs to produce the proinflammatory cytokines (**Fig. S4A**), and ∼1-fold stronger in sustaining the inflammatory cytokine production than LPS/CpG synergic stimulation (**Fig. S4B**). Collectively, these data indicate that expression of shS1/FliC possesses a unique ability to stimulate the prolonged and enhanced production of proinflammatory cytokines by DCs.

We next examined whether the shS1/FliC expression uniquely triggers and sustains TLR signaling cascades in DCs by comparing phosphorylation of key intracellular proinflammatory signaling molecules, such as STAT1 and STAT4, in Ad-shS1/FliC-transduced or TLR agonist-stimulated DCs. This is based upon that SOCS1 protein can be induced by TLR agonists, and SOCS1 overexpression inhibits JAK/STAT signaling triggered by a variety of cytokines in a negative feedback loop. Western Blot analyses revealed that higher levels of pSTAT1 and pSTAT4 were detected 72 hours after Ad-shS1/FliC-transduction of DCs compared with TLR agonist-stimulation of DCs, indicating the prolonged phosphorylation of the key inflammatory signaling molecules only occurs in the SOCS1-silenced DCs (**Fig. S5A**). Recent study indicated that SOCS1 is required for the rapid degradation of Mal, an adaptor protein involved in TLR2 and TLR4 signaling, to prevent Mal-dependent p65 phosphorylation and transactivation of NF-kB [24]. In support of the study, Western Blot analyses showed that, opposed to TLR agonist stimulation which led Mal to rapidly disappear (2 hr for LPS stimulation, 48 hr for Ad-FliC transduction), Mal was detected in DCs up to 96 hours post-transduction of Ad-shS1/FliC (24 hrs plus 72 hrs after wash) (**Fig. S5B**). Finally, we directly tested NF-kB activation in Ad-shS1-FliC-transduced BMDCs by monitoring the presence of NF-kB inhibitor ikBα. Western Blot assays showed that at 72 hrs after washout of the original stimulating or transducing media, Ad-shS1-FliC-transduced BMDCs exhibited a lower level of intracellular ikBα relative to LPS-stimulated BMDCs and a much lower level of ikBα relative to other control samples (**Fig. S5C**). Thus, these data suggest that the expression of shS1/FliC promotes the prolonged or enhanced production of proinflammatory cytokines likely by sustaining STAT signaling, TLR signaling, as well as downstream cytokine signaling cascades in DCs.

To elucidate whether the Ad-shS1/FliC responses observed above are specific for TLR5, BMDCs were transduced with Ad-shS1 followed by stimulation for 24 hrs with LPS, polyI:C, CpG, Imiquimod, purified FliC (Invivogen, San Diego, CA), or PBS at the indicated concentrations. Cytokine production from these treated BMDCs was compared with that from Ad-shS1/FliC-transduced BMDCs. We found that all of the TLR ligands promoted the inflammatory status of Ad-shS1-transduced BMDCs to different extents (concentrations of the TLR ligands used in the assays were not optimized), and LPS-mediated cytokine production (TNF-a, IL-12p40, and IL-6) from Ad-shS1-transduced BMDCs nearly reached the same levels of the cytokines produced by Ad-shS1/FliC-transduced BMDCs (**Fig. S6**). We also noted that although the recombinant FliC upregulated cytokine production from Ad-shS1-transduced BMDCs, the magnitude of the cytokines produced was significantly lower than that from Ad-shS1-FliC-transduced BMDCs (**Fig. S6**). The reason is possibly due to the FliC expressed by Ad-shS1/FliC is more native than the recombinant FliC with a tag sequence. Additionally, the purity and solubility of recombinant FliC in the culture media may contribute to the discrepancy. These results demonstrated that the observed Ad-shS1/FliC responses above are not specific for TLR5 and SOCS1 silencing boosts stimulant function of all the TLR ligands on BMDCs.

### The shS1/FliC-expressed DCs Exhibit Distinctive Epigenetic Modifications

**Figure 3:**
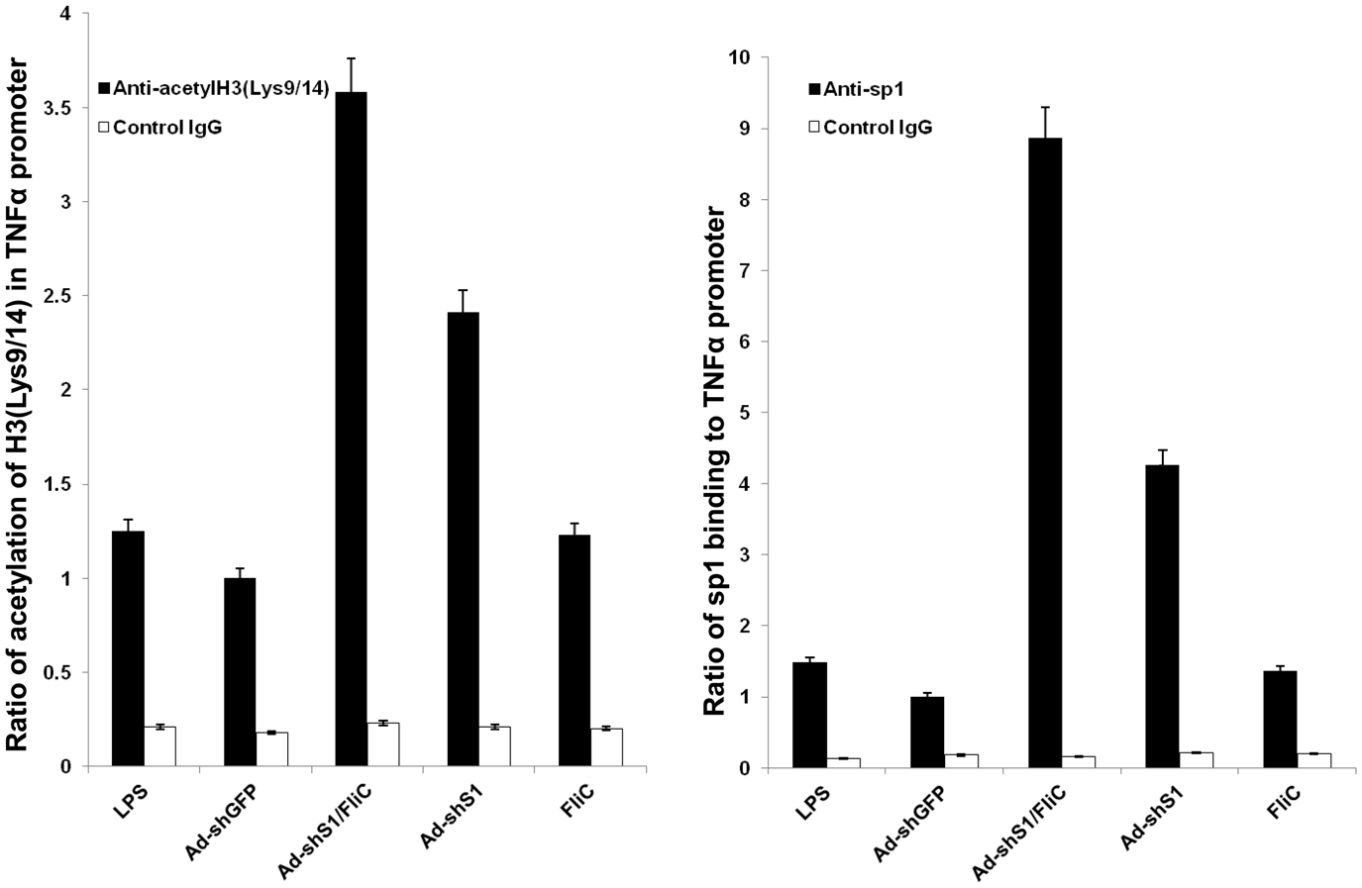
The expression of shS1/FliC enhances the acetylation of histone H3 at Lys9/14 and binding of transcription factor sp1 to the promoter of TNF-α in DCs. Murine BMDCs were transduced with the recombinant Ad vectors or stimulated with LPS (100 ng/ml). 24 h later, cultures were washed and replaced with fresh medium that contains LPS or not (for Ad-transduced DCs) for 72 hr. The cells were fixed with formaldehyde and a ChIP assay was performed using anti-acetyl histone H3 (Lys9/14) antibody (**A**) or anti-sp1 antibody (**B**). The precipitated DNA was amplified by real-time PCR using a pair of TNF-α promoter primers. The results were normalized to the input DNA and expressed as a ratio of Ad-shS1/FliC DC or LPS-stimulated DC to Ad-shGFP DC. *p<0.01.

Recent studies indicated that activation of cytokine transcription requires remodeling chromatin structure surrounding the promoter for recruitment of transcription factors and RNA polymerase II machinery [25], [26], [27]. Chromatin remodeling includes histone phosphorylation, acetylation and methylation, and the acetylation of histone H3 at lysine 9 and 14 (Lys9/14) in the promoter or vicinity is critical for gene activation. To investigate whether the proinflammatory status and duration of Ad-shS1/FliC DC is controlled by chromatin remodeling of proinflammatory cytokines, we performed Chromatin Immunoprecipitation **(**ChIP) assays to analyze a model inflammatory cytokine TNF-α on acetylation of histone H3 at Lys9/14 in the promoter region. Although no significant difference was detected on the histone H3 acetylation at the Lys9/14 of the TNF-α promoter when DCs were tested 24 hrs after Ad-shS1/FliC-transduction or LPS stimulation (**Fig. S7**), shS1/FliC-expressing DCs displayed ∼2-fold increase on the histone H3 acetylation at Lys9/14 compared with Ad-shGFP-transduced or LPS-stimulated DCs 72 hrs after culture or re-stimulation (**Fig. 3A**). In agreement with the histone H3 acetylation, Ad-shS1/FliC DCs also exhibited ∼6-fold increase in recruitment to the TNF-α promoter of the transcription factor sp1 (**Fig. 3B**), which was demonstrated to be important in LPS-stimulated TNF-α expression in several previous studies [28], [29]. We also found that histone H3 acetylation and sp1 recruitment to the TNF-α promoter in the Ad-shS1-transduced BMDCs were lower than those in the Ad-shS1/FliC-transduced BMDCs, but apparently higher than those in the LPS-stimulated BMDCs (**Fig. 3A&B**), implying that persistently inhibiting SOCS1 may be important in the chromatin remodeling. These results altogether supported that expression of shS1/FliC endows DCs with a superior ability to remodel chromatin structure of the TNF-α cytokine for sustaining the proinflammatory status.

### The shS1/FliC-expressed DCs Induce Potent Anti-HCV Cellular and Humoral Responses in Mice

**Figure 4:**
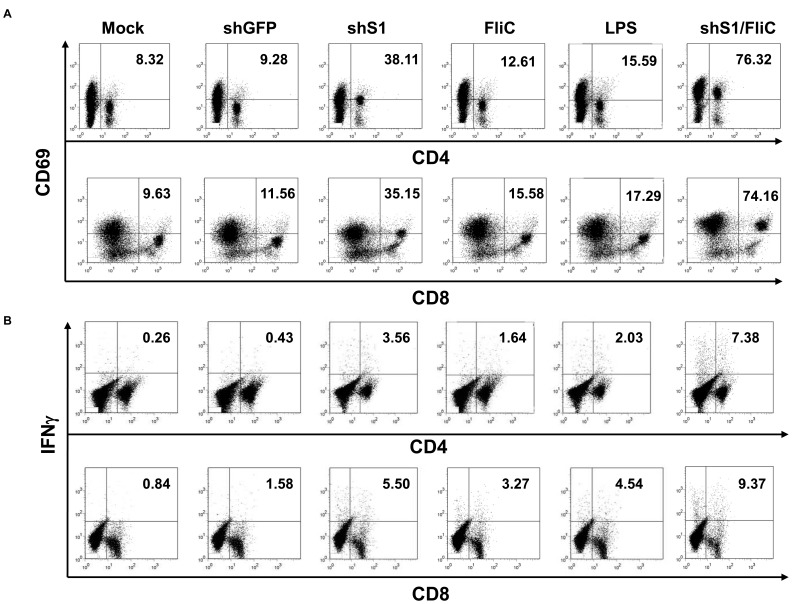
shS1/FliC-expressing DC immunization induces more potent HCV E2-specific T cell immune responses in mice. Murine BMDCs were transduced with the recombinant Ad vectors followed by pulse with recombinant HCV-E2 protein (20 µg/ml) for overnight, or pulsed with HCV-E2 for 6 hr prior to addition with LPS (100 ng/ml) and then cultured for overnight. Groups of C57BL/6 mice (6 mice/group) were immunized via footpads with the transduced or LPS-stimulated DCs (1×10^6^ cells per mouse) twice. 2 weeks after the 2^nd^ immunization, splenocytes were isolated for activation marker CD69 staining (**A**), and intracellular IFN-γ staining (**B**). Data are representative of three repeated experiments.

DC vaccination is an attractive means for controlling chronic infection, however, its potency and efficacy still need to be improved. To test the impact of the shS1/FliC expression on DC vaccination, groups of C57BL/6 mice were immunized via footpad with BMDCs that had been transduced with the adenoviral vectors or stimulated with LPS, the most potent of TLR agonists, and pulsed with a representative viral antigen HCV E2 that was produced and purified from a Baculovirus expression system with a purity of 95% [30]. Results showed that Ad-shS1/FliC-DCs were significantly more potent than DCs transduced with Ad-shS1 or Ad-FliC or stimulated with TLR agonists in inducing HCV E2-specific CD8^+^ cytotoxic T lymphocyte (CTL) and CD4^+^ T-helper (Th) responses, as demonstrated by the enhanced activation marker CD69 staining and intracellular IFNγ staining (**Fig. 4A–B** **)**. One representative of three experiments is shown with percentages of CD69- or IFNγ-positive CD4^+^ or CD8^+^ T cells from the individual mouse from the different immunization groups **(Fig. S8**). ELISPOT assays supported the superior potency of Ad-shS1/FliC-DCs in activating HCV E2-specific, IFNγ-producing CD8^+^ CTL and CD4^+^ Th (**Fig. S9**). In agreement with these results, qRT-PCR assay explored that the mRNA levels of Th1 transcription factor T-bet and Th2 transcription factors GATA-3 and c-Maf were significantly higher in CD4^+^ splenocytes from Ad-shS1/FliC DC immunization mice (**Fig. S10**).

**Figure 5:**
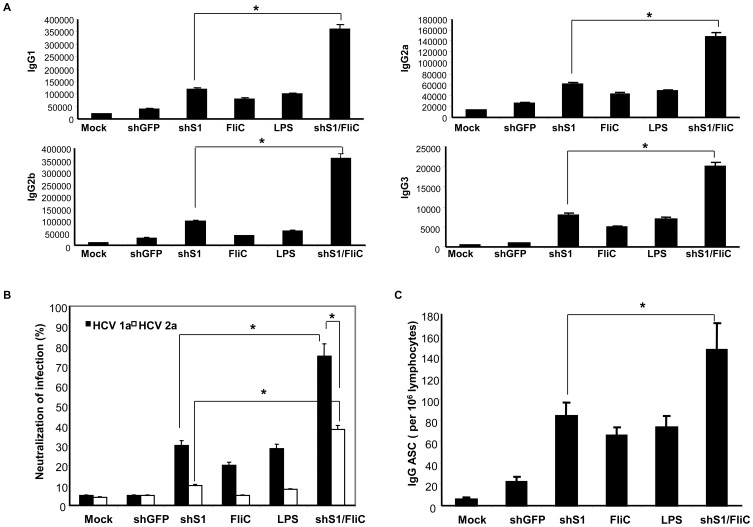
shS1/FliC-expressing DC immunization induces more potent HCV E2-specific humoral immune responses in mice. The above immunized mice were bled and sera were isolated for evaluation of HCV E2-specific IgG level by ELISA (**A**), and HCV E2-specific neutralizing antibody activity by HCVpp neutralization assay (**B**), splenocytes were isolated for evaluation of HCV E2-specific B cell response by ASC assay (**C**). Data are representative of three repeated experiments. *p<0.01 (Chi-square test).

We also tested Ad-shS1/FliC in potentiating DCs to induce anti-HCV antibody responses. **Fig. 5A** showed that an overall increase in HCV-E2-specific, different IgG antibody subsets in mice immunized with Ad-shS1/FliC-DCs compared with the mice immunized with DCs transduced with Ad-shS1 or Ad-FliC or stimulated with TLR agonists. To test if Ad-shS1/FliC-DCs induced a higher level of neutralizing antibody response, HCV E2-expressing retroviral pseudoparticle (HCVpp) was used to test neutralizing activity of the immunized sera as described previously [31]. **Fig. 5B** shows serum from Ad-shS1/FliC-DC mice efficiently neutralized ∼80% of the HCV-1a E2-expressing HCVpp, and crossly neutralized ∼40% of the HCV-2a E2-expressing HCVpp, owed to the conserved epitopes present across multiple HCV genotypes [32]. In contrast, either other Ad-transduced DCs or LPS-stimulated DCs only induced lower levels of neutralizing antibody response compared with Ad-shS1/FliC-DC immunization. To directly test the enhanced ability of Ad-shS1/FliC-DCs to activate B cells, Antibody Secretory Colony (ASC) assay was used to examine the frequencies of anti-HCV E2 IgG-producing B cells in the immunized spleens. **Fig. 5C** shows that frequencies of anti-HCV E2 IgG-producing B cells were significantly higher in Ad-shS1/FliC-DC mice than that in the immunized mice with DCs transduced with Ad-shS1 or Ad-FliC, or stimulated with TLR agonists. Thus, these results suggest that shS1/FliC-expressed DCs more effectively activate HCV E2-specific B cell response as well as neutralizing antibody response.

### The Expression of shS1/FliC Potently Activates Human Monocyte-derived DCs

**Figure 6:**
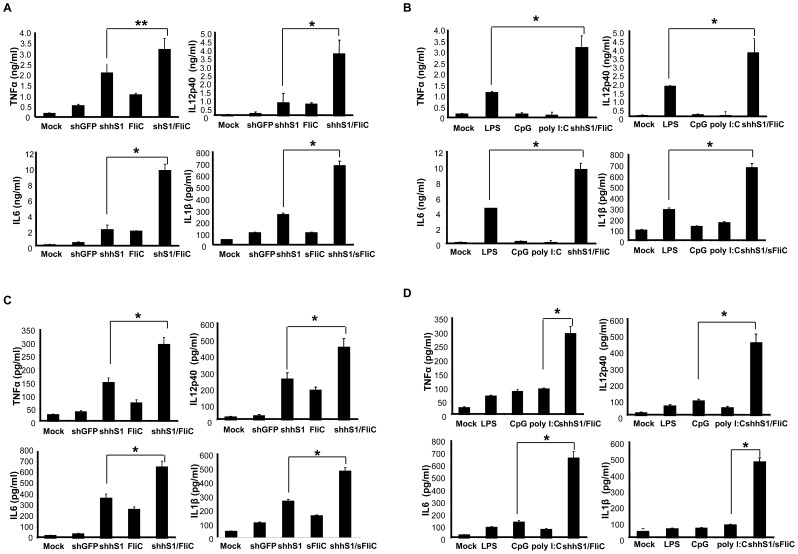
The expression of the human version shhS1/FliC potently activates human monocyte-derived DCs. Human monocyte-derived DCs were transduced with Ad vectors at an MOI of 250 or stimulated with different TLR agonists. **A & B.** 24 h later, culture media were collected from Ad-transduced DCs (**A**) or TLR-transduced DCs (**B**) for evaluation of the representative cytokines by ELISA. **C & D**. 24 h later, cultures were washed and replaced with fresh medium. The concentrations of representative cytokines in culture medium 3 days after the wash were examined by ELISA. Data are representative of three repeated experiments. *p<0.01, **p<0.05.

To assess the translational potential of the strategy, we tested whether shS1/FliC expression is also potent to stimulate human monocyte-derived DCs. Due to the heterogeneity of mouse and human SOCS1 sequences, we generated a recombinant vector Ad-shhS1/FliC that coexpresses FliC and a human (h) SOCS1-shRNA which is capable of efficiently silencing human SOCS1 mRNA expression (>80% reduction) (**Fig. S11A&B**). **Fig. 6A** shows that human DCs transduced with Ad-shhS1/FliC produced higher levels of the proinflammatory cytokines such as TNF-α, IL-12p40, IL-6, and IL-1β than human DCs transduced with Ad-shhS1 or Ad-FliC. We also found that human DCs transduced with Ad-shhS1/FliC produced significantly higher levels of the cytokines than DCs stimulated with the commonly used TLR agonists such as CpG, PolyI:C, or LPS (**Fig. 6B**). Furthermore, the duration of inflammatory cytokine production was significantly prolonged in human DCs transduced with Ad-shhS1/FliC compared to human DCs transduced with Ad-shhS1 or Ad-FliC, or stimulated with TLR agonists, as manifested by higher levels of proinflammatory cytokines were detected in Ad-shhS1/FliC transfected DCs at 24 hrs (**Fig. 6C**) and 72 hrs (**Fig. 6D**) after washing. These results indicate that SOCS1 controls the proinflammatory status and duration of human DCs, which is consistent with our previous study [18]. Thus, expression of human version shS1/FliC is more potent and persistent in stimulating monocyte-derived DCs than using the commonly used TLR agonist as an a maturation agent.

## Discussion

The present study provides evidences that combining TLR stimulation with inhibition of the negative regulation of proinflammatory signaling pathways may break the natural negative feedback barrier to trigger and sustain proinflammatoy signaling cascades/loops in APCs. Persistent and enhanced inflammatory signaling endows APCs with the unique ability to persistently activate innate and adaptive immunity. Indeed, the prototype generated from this concept, the super-TLR-agonist, is more potent than commonly used TLR agonists in stimulating the levels of proinflammatory cytokines produced by both murine and human DCs. Furthermore, expression of the super-TLR-agonist uniquely stimulates the production of inflammatory cytokines by DCs in a sustained manner. As a consequence, the super-agonist-expressed DCs have drastically enhanced potency in inducing both cellular and humoral immune responses against antigens of pathogens associated with chronic infections. Thus, expression of the super-agonist significantly facilitates DC vaccine to boost the magnitude of both cellular and humoral immune responses to higher levels that cannot be achieved by stimulation with TLR agonists or natural infections.

Persistent infections caused by pathogens such as HCV, HIV, hepatitis B virus (HBV), Epstein-Barr virus (EBV), and Tubercles Bacillus (TB) are major human diseases with limited or suboptimal therapeutic options. For example, chronic infection caused by HCV is characterized by weak cellular immune responses against viral antigens while viral clearance after acute hepatitis or after therapy is associated with strong and multispecific antiviral CD4 and CD8 T-cell responses [33], [34]. Therapeutic vaccination is a potentially useful strategy to control and eradicate persistent viral infections [35], [36], [37], [38]. Given the critical role of DCs in inducing immune responses, DC vaccination is an attractive means for therapeutic vaccination. However, the current therapeutic vaccines have been ineffective in the clinic. One of the reasons for the ineffectiveness may be owed to that these therapeutic vaccines do not elicit sufficient magnitude and duration of protective immune responses and they are unable to overcome Treg or pathogens-mediated immune suppression and evasion. Thus, expression of the super-agonist that is capable of enhancing the magnitude and duration of proinflammatory DCs and subsequent cellular and humoral responses may contribute to the induction of protective immunity in the chronically infectious hosts.

This study demonstrated the concept that the combined expression of a TLR agonist and an inhibitor of the negative regulation of proinflammatory signaling pathways in DCs can greatly promote the efficacy of DC vaccination. Adenoviral vectors may be suitable for ex vivo transduction of DCs despite that the high prevalence of human populations infected with certain adenoviral serotypes and adenovirus-associated immunogenicity may limit the use of adenoviruses as a delivery vehicle for in vivo administration [39], [40], [41]. Nevertheless, this concept could be generally applicable to create non-viral adjuvants for in vivo applications. For example, it is plausible to generate a flagellin-protamine (a DNA-binding protein) fusion protein to form complexes with SOCS1-siRNA as an adjuvant for activating APCs and adaptive immunity in vivo. Since the immune responses induced by vaccination or natural infection largely fail to effectively prevent and control persistent microbial infection, it is important to explore alternative immunization approaches and novel adjuvants to generate protective immune response that is superior to the natural immunity against persistent infections. The future studies will be focused on developing the super-TLR-agonist as an adjuvant to activate APCs and anti-viral immune responses in infectious or non-infectious hosts. Overall, the concept of the super-TLR-agonist capable of boosting and prolonging the TLR-triggering activation of DCs is novel and can be broadly used to improve the efficacy of DC vaccination in prevention and treatment of infectious diseases and tumors.

## Materials and Methods

### Mice

C57BL/6 mice were purchased from Jackson Laboratories and maintained in a pathogen-free mouse facility at USC according to institutional guidelines. This study was approved by the Institutional Animal Care and Use Committee of USC.

### Construction of Recombinant Adenoviruses

The AdEasy system was used to generate recombinant adenoviruses Ad-shS1/FliC, Ad-shhS1/FliC and control adenoviruses, as described in the Supplemental Materials (**Fig. S1**).

### Generation and Transduction of Mouse BMDCs and Human Monocyte-derived DCs with Ad-shS1/FliC

Mouse BMDCs were generated as previously described [42]. Immature DCs were harvested on day 5, resuspended in 500 ul RPMI 1640, added to 24-well plates and transduced with Ad-shS1/FliC or control adenovirus at an MOI of 250. Plates were spun for 90 min at 1000×g. DCs were cultured in RPMI 1640 supplemented with 10% FBS and 20 ng/ml GM-CSF and 20 ng/ml IL-4. Human monocyte-derived DCs were prepared as described [18]. Immature DCs were harvested and transduced with Ad-shhS1/FliC at a MOI of 250 as above. For cytokine production and duration assay, the transduced DCs were washed thoroughly and fed with new DC medium for another 24–72 hr.

### Western Blot Analysis

Mouse BMDCs were transduced with Ad-shS1/FliC or control adenovirus, or stimulated with LPS (100 ng/ml). Cell lysates were harvested 24–48 hr after transduction or stimulation. 20 µg of cell lysates were electrophoresed on SDS-PAGE, and electroblotted onto 0.22 µm nitrocellulose membrane (Hybond, AmershamBiosciences, Inc., Sunnyvale, CA). The membrane was then incubated with specific antibody and then Horseradish Peroxidase-conjugated second antibody (AmershamBiosciences, Inc., Sunnyvale, CA). The expressed protein was detected with ECL Plus Western Blotting detection kit (AmershamBiosciences, Inc., Sunnyvale, CA). Anti-FliC was purchased from Miravista Diagnostics (Indianapolis, IN). STAT1/pSTAT1, STAT4/pSTAT4 and Mal antibodies were purchased from Santa Cruz Biotechnology Inc. (Santa Cruz, CA). Anti-ikBα was purchased from Cell Signaling Inc. (Danvers, MA).

### DC Immunization

For DC immunization, the transduced DCs were pulsed with 20 µg/ml of HCV E2 protein [30] for overnight. The DCs were suspended in endotoxin-free PBS after washing. 5–6 week-old C57BL/6 mice were immunized via footpads with 1×10^6^ of DCs twice with a one-week interval. Two weeks after the 2nd immunization, the mice were sacrificed for evaluation of immune responses.

### ChIP Assay

Murine BMDCs were transduced with Ad-shS1/FliC or control Ad vectors at an MOI of 250 or stimulated with LPS (100 ng/ml). 24 h later, cultures were washed and replaced with fresh medium that contains LPS or not (for Ad-transduced DCs) for 72 hr. The cells were fixed with formaldehyde and a ChIP assay was perfrmed using anti-acetyl histone H3 (Lys9/14) (Cell Signaling) or anti-sp1(Santa Cruz Biotechnology) antibody. Assays were performed using the ChIP kit (Upstate) according to the manufacturer’s instructions. A pair of TNF-α promoter primers are the forward primer: 5′-CCCCAACTTTCCAAACCCTCT-3′; the reverse primer: 5′-CCCTCGGAAAACTTCCTTGGT-3′. The formula for quantitation of ChIP results is INPUT % = 1/9×2^Ct of INPUT − Ct of ChIP^ ×100.

### qRT-CR Analysis

For qRT-PCR analysis, the primers were used as below: mouse SOCS1 primers (forward, 5′-gagctgctggagcactacg-3′; reverse, 5′-agatctggaaggggaaggaa-3′), human SOCS1 primers (forward, 5′-cgacaatgcagtctccacag-3′; reverse, 5′-gaacggaatgtgcggaagt-3′), mouse T-bet (forward, 5′-tgttcccattcctgtccttc-3′; reverse, 5′-cccttgttgttggtgagctt-3′), mouse GATA-3 (forward,5′-ctggaggaggaacgctaatg-3′; reverse, 5′-cagggatgacatgtgtctgg-3′) and mouse c-Maf (forward, 5′-cactaccatcaccaccacca-3′; reverse, 5′-gagaagcggtcgtcgaagt-3′).

### Flow Cytometry Analysis

For analysis of mouse DCs, fluorescein isothiocyanate (FITC)-, phycoerythrin (PE)- or allophycocyanin (APC)-conjugated monoclonal antibodies (Mabs) against mouse CD40 (5C-3), CD80 (3H5), CD86 (GL1), I-A/I-E (M5/114.15.2) and matched isotype controls were used for multiple color staining. For analysis of T cells, FITC-, PE-, or APC- conjugated MAbs against mouse CD4 (GK1.5), CD8 (OX-8), or IFN- γ (XMG1.2) were used. All these Mabs were purchased from BD Bioscience.

### ELISPOT

ELISPOT assays were performed as described previously [43]. Briefly, MultiScreen-HA plates (Millipore, Bedford, MA) were incubated with anti-mouse-IFN-γ mAb AN18 (Mabtech, Stockholm, Sweden) overnight, and then blocked with RPMI 1640 supplemented with 10% FBS (Bethyl, Inc., Montgomery, TX). Splenocytes from immunized mice were cultured at 2×10^5^/well in triplicate in the presence of 10 µg/ml HCV E2 protein in the coated plate for 20 hr at 37°C. After washing, biotinylated anti-mouse IFNγ antibody R4-6A2 (Mabtech) was added to the wells, and incubated for 2 hr. After another wash, HRP-conjugated avidin was added to the wells and incubated for 1 hr. Spots were developed by the addition of HRP substrate (Vectastain ABC Kit, Vector Laboratories).

### ELISA

Proinflammatory cytokines were quantitated from the supernatant of DC cultures by ELISA (BD Biosciences) according to the manufacturer’s instructions. Antibody levels in immunized mice were determined by ELISA. 50 ng of HCV E2 protein was used to coat microtiter plates (BD Biosciences, Oxnard, CA).

### ASC Assay

Single-cell suspensions were prepared from spleens of immunized mice. MultiScreen-HA plates were coated with 0.5 ug of HCV E2 protein for overnight and blocked at 37°C for 2 hours. A varying number of splenocytes (5×10^4^ to 1×10^6^) were added to the wells, and the plates were incubated at 37°C for overnight. Cells were removed, and the plates were washed extensively. For detection of secreted antibody, 100 µl of HRP-conjugated goat anti-mouse IgG (1 µg/ml) (Santa Cruz Biotechnology) was added to the wells, and the plates were incubated overnight at 4°C. After another wash, the wells were added with 100 µl of 3-amino-9-ethylcarbazole (Sigma) to allow spot development (∼4 min) before the reaction was stopped with H_2_O. Antibody secretory colonies were enumerated under Stereozoom 5 dissecting microscope (Leica, Buffalo, NY).

### HCV Neutralization Assay

Neutralization assay was performed as described [31]. The HCV E2-expressing retroviral pseudoparticle (HCVpp) was engineered to express a luciferase gene [44]. To test neutralizing activity of immunized sera, the HCVpp inoculum was incubated with the diluted sera (1∶100) for 1 hour at room temperature prior to inoculation of Huh-7 cell culture. The HCVpp infection of Huh-7 cell proceeded for 8 hours at 37°C. The infected Huh-7 cells were changed with fresh medium and incubated for another 72 hours. The cell lysates were prepared for luciferase assay (Promega). Neutralization activity was expressed as the percentage of the immunized serum reducing luciferase expression by HCVpp in comparison with normal serum did.

### Statistical Analysis

For statistical analysis, we used Student’s t test or Chi-square test (indicated when being used), and a 95% confidence limit was taken to be significant, defined as *p*<0.05. Results are typically presented as means ± standard errors (SE).

## Supporting Information

Figure S1 **Construction and characterization of recombinant Ad vectors.** **A**, schematic representation of recombinant Ad vectors**.** The AdEasy system was used to construct and generate replication-defective adenoviruses. The FliC gene was amplified by PCR using *Salmonella enterica serovar Typhimurium* (ATCC) as a template. For facilitating the secretion and subsequent interaction with surface TLR5 on DCs, a signal leader sequence derived from human tyrosinase was genetically linked to the N-terminus of the FliC gene by PCR with a pair of primers (5′- TAGTCGACCTCGAGATGCTCCTGGCTGTTTTGTACTGCCTGCTGTGGAGTTTCCAGACCTCCGCTGGCCATTTCCCTAGAATGGCACAAGTCATTA-3′ and 5′-GGCTCTAGAGCGGCCGCTTAACGCAGTAAAGAGAGG-3′). The mouse shS1 (CTACCTGAGTTCCTTCCCCTT) or human shS1 (hshS1, CACGCACTTCCGCACATTC) with a U6 promoter at the upstream was inserted into the Ad vector, and the resultant Ad vectors were confirmed by DNA sequencing. **B & C**, BMDCs were transduced with Ad-shS1/FliC or control Ad vectors at an MOI of 250 for 24 hr. mRNA level of SOCS1 were analyzed by qRT-PCR. *p<0.01 (**B**). Cell lysates were prepared and FliC expression was tested by Western Blot (**C**).
(TIF)

Figure S2 **Surface expression of TLR5 on D2SC1, J774 and BMDC.** Exponentially growing D2SC1 and J774, and differentiated BMDCs were stained with PE-conjugated anti-TLR5 (IMGENEX, San Diego, CA) for flow cytometry analysis.
(TIF)

Figure S3 **Cytokine production by differently treated J774 cells.** Murine J774 cells were transduced with the recombinant Ad vectors at an MOI of 250. 24 h later, culture media were collected for evaluation of the representative cytokines by ELISA. Data are representative of three repeated experiments. *p<0.05.
(TIF)

Figure S4 **Expression of shS1/FliC is superior to TLR synergic stimulation in inducing inflammatory status and duration of BMDCs.** Murine BMDCs were transduced with Ad-shS1/FliC at an MOI of 250 or stimulated with commonly used TLR agonists or LPS/CpG synergy. **A.** 24 hr later, culture media were collected for evaluation of the representative cytokines by ELISA. **B,** 24 hr later, cultures were washed and replaced with fresh medium. Concentrations of the representative cytokines in culture media 3 days after the wash were examined by ELISA. Data are representative of three repeated experiments. *p<0.01.
(TIF)

Figure S5 **The shS1/FliC-expressed DCs display enhanced TLR and downstream cytokine signaling.** Murine BMDCs were transduced with Ad-shS1/FliC or control Ad vectors at an MOI of 250 or stimulated with LPS (100 ng/ml). 24 h later, cultures were washed and replaced with fresh medium. **A.** Cell lysates were prepared 72 hrs after the wash and subject to Western Blot analysis of STAT1/pSTAT1, STAT4/pSTAT4. **B**. Cell lysates were prepared at 0, 2, and 24 hr after transduction or stimulation, or 24 and 72 hr after the washout. The cell lysates were subject to Western Blot analysis of Mal degradation. **C**. Cell lysates were prepared at 72 hr after the washout and subject to Western Blot for analysis of ikBα expression.
(TIF)

Figure S6 **TLR ligands stimulate Ad-shS1-transduced BMDCs.** Murine BMDCs were transduced with Ad-shS1/FliC or with Ad-shS1 followed by stimulation with LPS (100 ng/ml), CpG (1 µM), PolyI:C (1 µg/ml), Imiquimod (2 µg/ml), FliC (2 µg/ml), or PBS. 24 h later, culture media were harvested to analyze expression of the representative cytokines by ELISA. Ad-shS1/LPS vs. Ad-shS1/FliC is not statistically significant.
(TIF)

Figure S7 **Acetylation of histone H3 at Lys9/14 in the promoter of TNF-α** **in differently treated BMDCs.** Murine BMDCs were transduced with the recombinant Ad vectors or stimulated with LPS (100 ng/ml). 24 h later, the cells were fixed with formaldehyde and a ChIP assay was performed using anti-acetyl histone H3 (Lys9/14) antibody. The precipitated DNA was amplified by real-time PCR using a pair of TNF-α promoter primers. The results were normalized to the input DNA and expressed as a ratio of Ad-shS1/FliC DC or LPS-stimulated DC to Ad-shGFP DC.
(TIF)

Figure S8 **shS1/FliC-expressing DC immunization induced high percentages of HCV E2-specific CD69^+^− or IFNγ^+^−T cells.** Murine BMDCs were transduced with the recombinant Ad vectors followed by pulse with recombinant HCV-E2 protein (20 µg/ml) for overnight, or pulsed with HCV-E2 for 6 hr prior to addition with LPS (100 ng/ml) and then cultured for overnight. Groups of C57BL/6 mice (6 mice/group) were immunized via footpads with the transduced or LPS-stimulated DCs (1×10^6^ cells per mouse) twice. 2 weeks after the 2^nd^ immunization, splenocytes were isolated for activation marker CD69 staining (**A**), and intracellular IFN-γ staining (**B**). Data are expressed as the percentages of CD69- or IFNγ-positive CD4^+^ or CD8^+^ T cells from the individual mouse of the differently immunized groups and a representative of three repeated experiments.
(TIF)

Figure S9 **Immunization with shS1/FliC-expressing DCs enhances the frequency of T cells producing IFN-γ in response to HCV E2 stimulation.** Murine BMDCs were transduced with recombinant Ad vectors at an MOI of 250 followed by pulse with recombinant HCV-E2 protein (20 µg/ml) for overnight, or pulsed with HCV-E2 for 6 hr prior to addition with LPS (100 ng/ml) and then cultured for overnight. Groups of C57BL/6 mice were immunized via footpads with the transduced or LPS-stimulated DCs (1×10^6^ cells per mouse) twice at a weekly interval. 2 wks after the 2^nd^ immunization, mice were sacrificed, splenocytes were isolated for ELISPOT assay. The experiment was repeated twice. *p<0.01.
(TIF)

Figure S10 **Immunization with shS1/FliC-expressing DCs enhances expression of transcription factors T-bet, GATA-3, and cMaf in CD4^+^ T cells.** CD4^+^ cells were purified from splenocytes of Ad-shS1/sFliC DC immunized or control mice. T-bet, GATA-3 and c-Maf expression was analyzed by qRT-PCR with the corresponding primers. *p<0.01.
(TIF)

Figure S11 **Characterization of human version Ad-shhS1/FliC.** Monocyte-derived DCs were transduced with Ad-shhS1/FliC at an MOI of 0, 1.0, 10, 100, or 250 (lane 1–5) for 24 hr. **A,** supernatants were harvested (**the left panel**) and cell lysates were prepared (**the right panel**) for analysis of FliC expression and secretion by Western Blot. **B,** Monocyte-derived DCs were transduced with Ad-shhS1/FliC or control adenovirus Ad-shGFP, Ad-shhS1 or Ad-FliC at an MOI of 250 for 24 hr. Total cellular RNA was extracted and mRNA level of SOCS1 was analyzed by qRT-PCR. *p<0.01.
(TIF)

## References

[1] Banchereau J, Steinman RM (1998) Dendritic cells and the control of immunity. Nature 392: 245–252. 9521319 10.1038/32588

[2] Akira S, Takeda K (2004) Toll-like receptor signalling. Nat Rev Immunol 4: 499–511. 15229469 10.1038/nri1391

[3] Kanzler H, Barrat FJ, Hessel EM, Coffman RL (2007) Therapeutic targeting of innate immunity with Toll-like receptor agonists and antagonists. Nat Med 13: 552–559. 17479101 10.1038/nm1589

[4] Yoshimura A, Naka T, Kubo M (2007) SOCS proteins, cytokine signalling and immune regulation. Nat Rev Immunol 7: 454–465. 17525754 10.1038/nri2093

[5] Shen L, Evel-Kabler K, Strube R, Chen SY (2004) Silencing of SOCS1 enhances antigen presentation by dendritic cells and antigen-specific anti-tumor immunity. Nat Biotechnol 22: 1546–1553. 15558048 10.1038/nbt1035

[6] Song X-T, Kabler KE, Shen L, Rollins L, Huang XF, et al (2008) A20 is an antigen presentation attenuator, and its inhibition overcomes regulatory T cell-mediated suppression. Nat Med 14: 258–265. 18311150 10.1038/nm1721 PMC3684168

[7] Hayashi F, Smith KD, Ozinsky A, Hawn TR, Yi EC, et al (2001) The innate immune response to bacterial flagellin is mediated by Toll-like receptor 5. Nature 410: 1099–1103. 11323673 10.1038/35074106

[8] Means TK, Hayashi F, Smith KD, Aderem A, Luster AD (2003) The Toll-Like Receptor 5 Stimulus Bacterial Flagellin Induces Maturation and Chemokine Production in Human Dendritic Cells. J Immunol 170: 5165–5175. 12734364 10.4049/jimmunol.170.10.5165

[9] Cunningham AF, Khan M, Ball J, Toellner KM, Serre K, et al (2004) Responses to the soluble flagellar protein FliC are Th2, while those to FliC on Salmonella are Th1. Eur J Immunol 34: 2986–2995. 15384042 10.1002/eji.200425403

[10] Didierlaurent A, Ferrero I, Otten LA, Dubois B, Reinhardt M, et al (2004) Flagellin promotes myeloid differentiation factor 88-dependent development of Th2-type response. J Immunol 172: 6922–6930. 15153511 10.4049/jimmunol.172.11.6922

[11] McSorley SJ, Ehst BD, Yu Y, Gewirtz AT (2002) Bacterial flagellin is an effective adjuvant for CD4+ T cells in vivo. J Immunol 169: 3914–3919. 12244190 10.4049/jimmunol.169.7.3914

[12] Cuadros C, Lopez-Hernandez FJ, Dominguez AL, McClelland M, Lustgarten J (2004) Flagellin fusion proteins as adjuvants or vaccines induce specific immune responses. Infect Immun 72: 2810–2816. 15102791 10.1128/IAI.72.5.2810-2816.2004 PMC387897

[13] Huleatt JW, Jacobs AR, Tang J, Desai P, Kopp EB, et al (2007) Vaccination with recombinant fusion proteins incorporating Toll-like receptor ligands induces rapid cellular and humoral immunity. Vaccine 25: 763–775. 16968658 10.1016/j.vaccine.2006.08.013

[14] Lee SE, Kim SY, Jeong BC, Kim YR, Bae SJ, et al (2006) A bacterial flagellin, Vibrio vulnificus FlaB, has a strong mucosal adjuvant activity to induce protective immunity. Infect Immun 74: 694–702. 16369026 10.1128/IAI.74.1.694-702.2006 PMC1346682

[15] Applequist SE, Rollman E, Wareing MD, Liden M, Rozell B, et al (2005) Activation of innate immunity, inflammation, and potentiation of DNA vaccination through mammalian expression of the TLR5 agonist flagellin. J Immunol 175: 3882–3891. 16148134 10.4049/jimmunol.175.6.3882

[16] Yang TC, Millar JB, Grinshtein N, Bassett J, Finn J, et al (2007) T-cell immunity generated by recombinant adenovirus vaccines. Expert Review of Vaccines 6: 347–356. 17542750 10.1586/14760584.6.3.347

[17] Mossoba ME, Medin JA (2006) Cancer immunotherapy using virally transduced dendritic cells: animal studies and human clinical trials. Expert Review of Vaccines 5: 717–732. 17181444 10.1586/14760584.5.5.717

[18] Hong B, Ren W, Song XT, Evel-Kabler K, Chen SY, et al (2009) Human suppressor of cytokine signaling 1 controls immunostimulatory activity of monocyte-derived dendritic cells. Cancer Res 69: 8076–8084. 19789342 10.1158/0008-5472.CAN-09-1507 PMC2763040

[19] Iezzi G, Karjalainen K, Lanzavecchia A (1998) The Duration of Antigenic Stimulation Determines the Fate of Naive and Effector T Cells. Immunity 8: 89–95. 9462514 10.1016/s1074-7613(00)80461-6

[20] van Duin D, Medzhitov R, Shaw AC (2006) Triggering TLR signaling in vaccination. Trends in Immunology 27: 49–55. 16310411 10.1016/j.it.2005.11.005

[21] Breckpot K, Aerts-Toegaert C, Heirman C, Peeters U, Beyaert R, et al (2009) Attenuated expression of A20 markedly increases the efficacy of double-stranded RNA-activated dendritic cells as an anti-cancer vaccine. J Immunol 182: 860–870. 19124729 10.4049/jimmunol.182.2.860

[22] Evel-Kabler K, Song XT, Aldrich M, Huang XF, Chen SY (2006) SOCS1 restricts dendritic cells’ ability to break self tolerance and induce antitumor immunity by regulating IL-12 production and signaling. J Clin Invest 116: 90–100. 16357940 10.1172/JCI26169 PMC1312019

[23] Napolitani G, Rinaldi A, Bertoni F, Sallusto F, Lanzavecchia A (2005) Selected Toll-like receptor agonist combinations synergistically trigger a T helper type 1-polarizing program in dendritic cells. Nat Immunol 6: 769–776. 15995707 10.1038/ni1223 PMC3760217

[24] Mansell A, Smith R, Doyle SL, Gray P, Fenner JE, et al (2006) Suppressor of cytokine signaling 1 negatively regulates Toll-like receptor signaling by mediating Mal degradation. Nat Immunol 7: 148–155. 16415872 10.1038/ni1299

[25] Garrett S, Dietzmann-Maurer K, Song L, Sullivan KE (2008) Polarization of primary human monocytes by IFN-gamma induces chromatin changes and recruits RNA Pol II to the TNF-alpha promoter. J Immunol 180: 5257–5266. 18390706 10.4049/jimmunol.180.8.5257

[26] Lee JY, Kim NA, Sanford A, Sullivan KE (2003) Histone acetylation and chromatin conformation are regulated separately at the TNF-alpha promoter in monocytes and macrophages. J Leukoc Biol 73: 862–871. 12773519 10.1189/jlb.1202618

[27] Sullivan KE, Reddy AB, Dietzmann K, Suriano AR, Kocieda VP, et al (2007) Epigenetic regulation of tumor necrosis factor alpha. Mol Cell Biol 27: 5147–5160. 17515611 10.1128/MCB.02429-06 PMC1951949

[28] Cooper ZA, Singh IS, Hasday JD (2010) Febrile range temperature represses TNF-alpha gene expression in LPS-stimulated macrophages by selectively blocking recruitment of Sp1 to the TNF-alpha promoter. Cell Stress Chaperones 15: 665–673. 20221720 10.1007/s12192-010-0179-9 PMC3006616

[29] Sandoval J, Pereda J, Rodriguez JL, Escobar J, Hidalgo J, et al (2010) Ordered transcriptional factor recruitment and epigenetic regulation of tnf-alpha in necrotizing acute pancreatitis. Cell Mol Life Sci 67: 1687–1697. 20130956 10.1007/s00018-010-0272-3 PMC11115704

[30] Machida K, Cheng KT, Pavio N, Sung VM, Lai MM (2005) Hepatitis C virus E2-CD81 interaction induces hypermutation of the immunoglobulin gene in B cells. J Virol 79: 8079–8089. 15956553 10.1128/JVI.79.13.8079-8089.2005 PMC1143751

[31] Bartosch B, Bukh J, Meunier JC, Granier C, Engle RE, et al (2003) In vitro assay for neutralizing antibody to hepatitis C virus: evidence for broadly conserved neutralization epitopes. Proc Natl Acad Sci U S A 100: 14199–14204. 14617769 10.1073/pnas.2335981100 PMC283569

[32] Law M, Maruyama T, Lewis J, Giang E, Tarr AW, et al (2008) Broadly neutralizing antibodies protect against hepatitis C virus quasispecies challenge. Nat Med 14: 25–27. 18064037 10.1038/nm1698

[33] Missale G, Bertoni R, Lamonaca V, Valli A, Massari M, et al (1996) Different clinical behaviors of acute hepatitis C virus infection are associated with different vigor of the anti-viral cell-mediated immune response. The Journal of Clinical Investigation 98: 706–714. 8698862 10.1172/JCI118842 PMC507480

[34] Thimme R, Oldach D, Chang K-M, Steiger C, Ray SC, et al (2001) Determinants of Viral Clearance and Persistence during Acute Hepatitis C Virus Infection. J Exp Med 194: 1395–1406. 11714747 10.1084/jem.194.10.1395 PMC2193681

[35] Moingeon P, Almond J, de Wilde M (2003) Therapeutic vaccines against infectious diseases. Current Opinion in Microbiology 6: 462–471. 14572538 10.1016/j.mib.2003.08.002

[36] Autran B, Carcelain G, Combadiere B, Debre P (2004) Therapeutic Vaccines for Chronic Infections. Science 305: 205–208. 15247470 10.1126/science.1100600

[37] Lu W, Arraes LC, Ferreira WT, Andrieu JM (2004) Therapeutic dendritic-cell vaccine for chronic HIV-1 infection. Nat Med 10: 1359–1365. 15568033 10.1038/nm1147

[38] Nevens F, Roskams T, Vlierberghe HV, Horsmans Y, Sprengers D, et al (2003) A pilot study of therapeutic vaccination with envelope protein E1 in 35 patients with chronic hepatitis C. Hepatology. 38: 1289–1296. 10.1053/jhep.2003.50474 14578869

[39] Thomas CE, Ehrhardt A, Kay MA (2003) Progress and problems with the use of viral vectors for gene therapy. Nat Rev Genet 4: 346–358. 12728277 10.1038/nrg1066

[40] Perreau M, Pantaleo G, Kremer EJ (2008) Activation of a dendritic cell-T cell axis by Ad5 immune complexes creates an improved environment for replication of HIV in T cells. J Exp Med 205: 2717–2725. 18981239 10.1084/jem.20081786 PMC2585831

[41] Zhu J, Huang X, Yang Y (2007) Innate immune response to adenoviral vectors is mediated by both Toll-like receptor-dependent and -independent pathways. J Virol 81: 3170–3180. 17229689 10.1128/JVI.02192-06 PMC1866082

[42] Prasad SJ, Farrand KJ, Matthews SA, Chang JH, McHugh RS, et al (2005) Dendritic cells loaded with stressed tumor cells elicit long-lasting protective tumor immunity in mice depleted of CD4+CD25+ regulatory T cells. J Immunol 174: 90–98. 15611231 10.4049/jimmunol.174.1.90

[43] Song XT, Evel-Kabler K, Shen L, Rollins L, Huang XF, et al (2008) A20 is an antigen presentation attenuator, and its inhibition overcomes regulatory T cell-mediated suppression. Nat Med 14: 258–265. 18311150 10.1038/nm1721 PMC3684168

[44] Machida K, Kondo Y, Huang JY, Chen YC, Cheng KT, et al (2008) Hepatitis C virus (HCV)-induced immunoglobulin hypermutation reduces the affinity and neutralizing activities of antibodies against HCV envelope protein. J Virol 82: 6711–6720. 18417597 10.1128/JVI.02582-07 PMC2447061

