# Supplementary figures and images for: A Super TLR Agonist to Improve Efficacy of Dendritic Cell Vaccine in Induction of Anti-HCV Immunity
Source: PLoS One. 2012 Nov 7;7(11):e48614. doi: 10.1371/journal.pone.0048614 (PMC3492467; doi:10.1371/journal.pone.0048614)

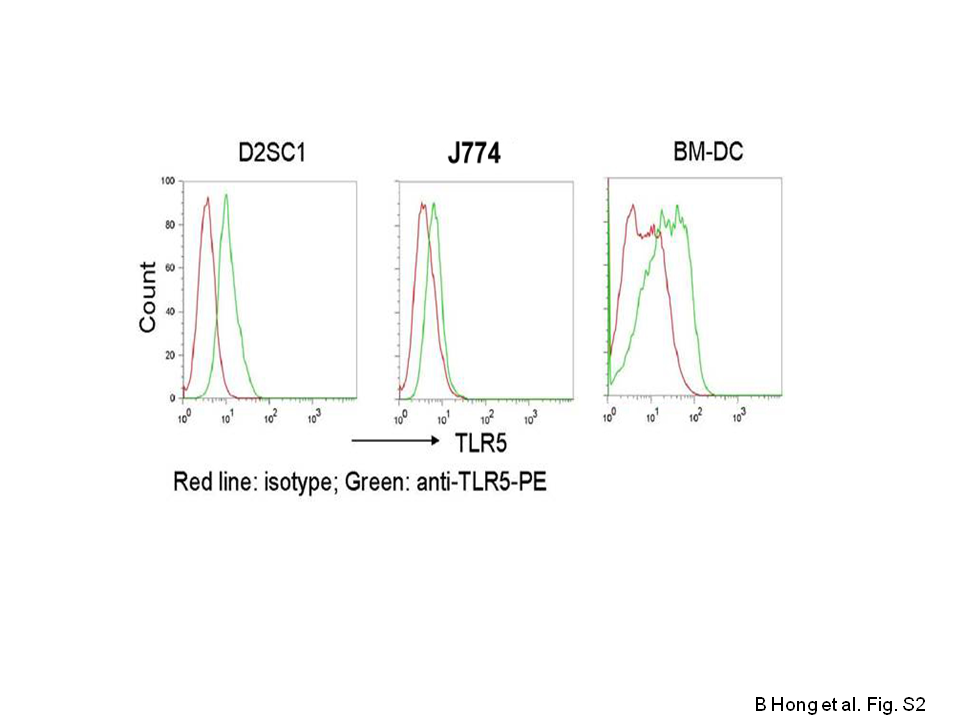

Supplement: Figure S2 — Surface expression of TLR5 on D2SC1, J774 and BMDC. Exponentially growing D2SC1 and J774, and differentiated BMDCs were stained with PE-conjugated anti-TLR5 (IMGENEX, San Diego, CA) for flow cytometry analysis. (TIF) [file pone.0048614.s002.tif]

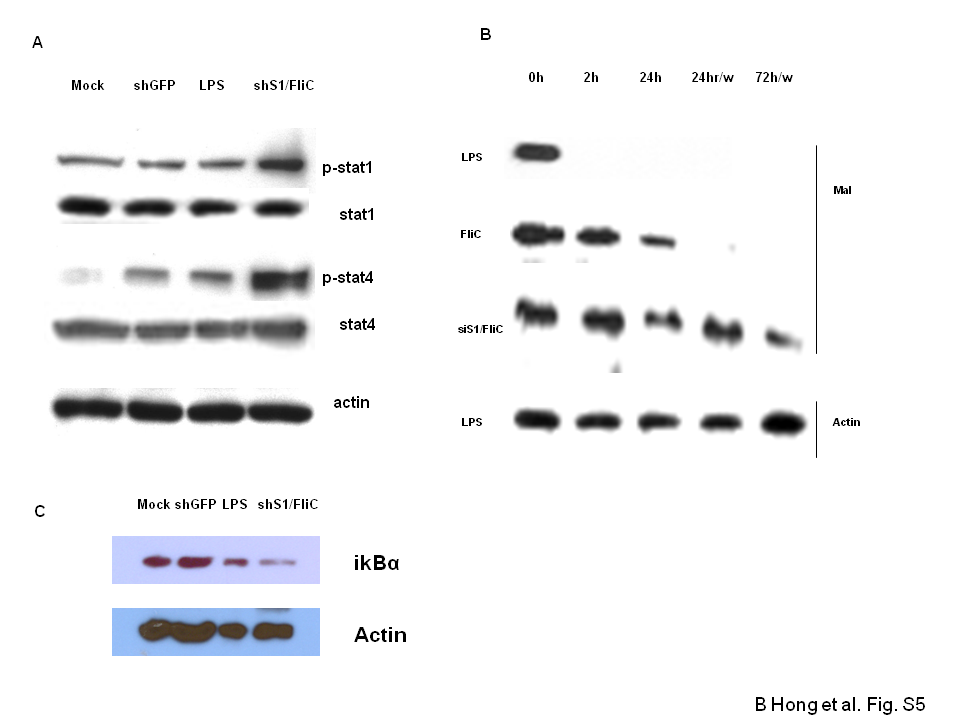

Supplement: Figure S5 — The shS1/FliC-expressed DCs display enhanced TLR and downstream cytokine signaling. Murine BMDCs were transduced with Ad-shS1/FliC or control Ad vectors at an MOI of 250 or stimulated with LPS (100 ng/ml). 24 h later, cultures were washed and replaced with fresh medium. A. Cell lysates were prepared 72 hrs after the wash and subject to Western Blot analysis of STAT1/pSTAT1, STAT4/pSTAT4. B. Cell lysates were prepared at 0, 2, and 24 hr after transduction or stimulation, or 24 and 72 hr after the washout. The cell lysates were subject to Western Blot analysis of Mal degradation. C. Cell lysates were prepared at 72 hr after the washout and subject to Western Blot for analysis of ikBα expression. (TIF) [file pone.0048614.s005.tif]

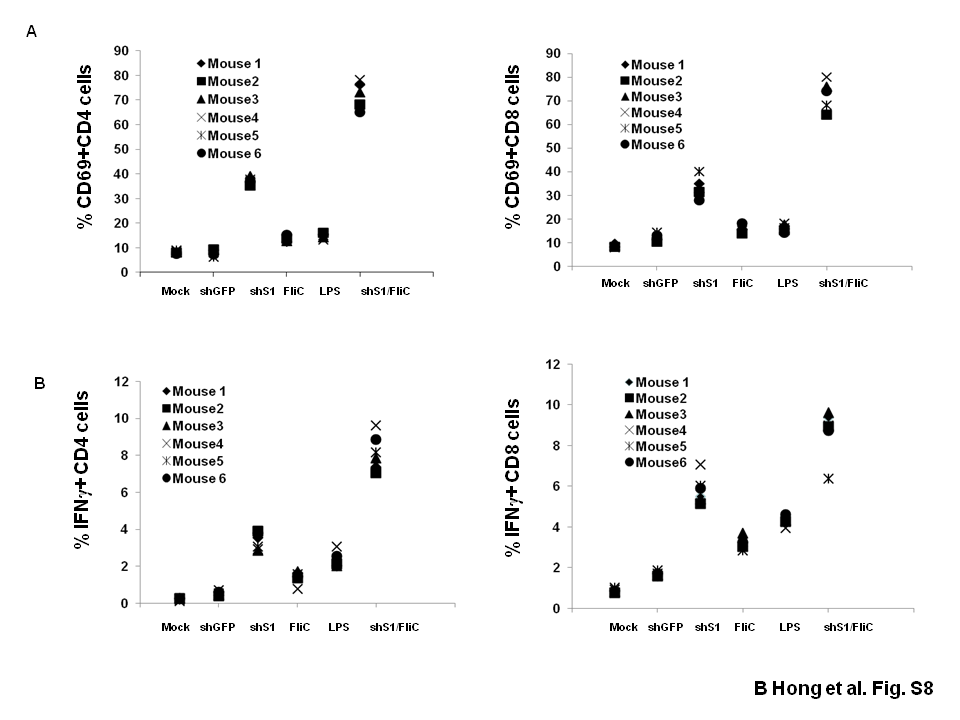

Supplement: Figure S8 — shS1/FliC-expressing DC immunization induced high percentages of HCV E2-specific CD69+− or IFNγ+−T cells. Murine BMDCs were transduced with the recombinant Ad vectors followed by pulse with recombinant HCV-E2 protein (20 µg/ml) for overnight, or pulsed with HCV-E2 for 6 hr prior to addition with LPS (100 ng/ml) and then cultured for overnight. Groups of C57BL/6 mice (6 mice/group) were immunized via footpads with the transduced or LPS-stimulated DCs (1×10^6^ cells per mouse) twice. 2 weeks after the 2nd immunization, splenocytes were isolated for activation marker CD69 staining (A), and intracellular IFN-γ staining (B). Data are expressed as the percentages of CD69- or IFNγ-positive CD4+ or CD8+ T cells from the individual mouse of the differently immunized groups and a representative of three repeated experiments. (TIF) [file pone.0048614.s008.tif]
